# Correction for non-uniform k-space data weighting effects in first-pass cardiac perfusion imaging with TurboFLASH readout

**DOI:** 10.1186/1532-429X-14-S1-P278

**Published:** 2012-02-01

**Authors:** Sohae Chung, Leon Axel

**Affiliations:** 1 Radiology, NYU Langone Medical Center, New York, NY, USA

## Summary

To correct for non-uniform k-space data weighing on image intensity in T1-weighted first-pass cardiac perfusion MR imaging with TurboFLASH readout by using numerical simulations.

## Background

To obtain first-pass cardiac perfusion images, a saturation-recovery (SR) preparation can be used with TurboFLASH readout. However, non-uniform k-space weighting during the SR recovery may lead to distortion of the image point spread function; this may lead to systematic overestimation or underestimation of the image-derived arterial input function (AIF) and myocardium signals, with resulting bias in the perfusion calculations. In this work, we used numerical simulations to correct for non-uniform k-space data weighting effects on the AIF and myocardial wall signals.

## Methods

First-pass perfusion MRI was performed in six healthy volunteers (29±12 y.o.; 3T MR scanner, Tim Trio, Siemens). Images were obtained at 2 slice locations (the aortic root for the AIF and the basal level of the left ventricle for the wall; Fig.1a) using a SR TurboFLASH readout with centric k-space reordering in order to minimize the sensitivity to inflow effects [1]. Imaging parameters included [2]: FOV=350mm×315mm, slice thickness=8mm, image matrix=160×144, in-plane resolution=2.2mm×2.2mm, TE/TR=1.2/2.4ms, TD(AIF/wall)=50/164ms, flip angle=10°, and receiver bandwidth=1008Hz/pixel. A proton density-weighted image was acquired for normalization [3]. For signal correction, a numerical model of each representative object geometry was generated and the corresponding T1-weighted signals in k-space were calculated, using the Bloch equation [3] with the same parameters used in MR imaging. A look-up table (Fig.1b) was created to show the relationship between the image signals with and without non-uniform k-space data weighting for the AIF and wall; this was used to correct the observed AIF and wall image signals.

**Figure 1:**
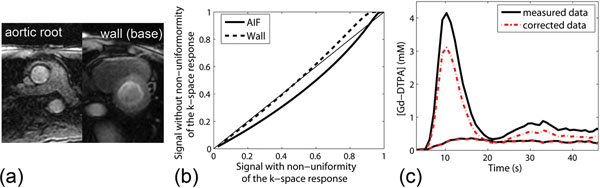
(a) Representative perfusion MR images at (left) the aortic root and (right) short-axis base. (b) A look-up table for AIF and wall. (c) Measured and corrected Gd-DTPA concentration of the AIF and wall.

## Results

After signal correction using the calculated table, reduced signals and corresponding calculated Gd-DTPA concentrations (Fig.1c) were found in both AIF and the wall. The average percent differences between the measured and corrected values of the signals and Gd-DTPA concentrations for all subjects were 18.4±5.7% and 19.7±5.5% for the AIF, and 2.6±1.1% and 2.1±1.0% for the wall, respectively.

## Conclusions

We have found that non-uniform k-space weighting in centric SR primarily affects the AIF, with less effect on the signal in the myocardial wall. Thus, it is important to correct for these differences, in order to avoid resulting systematic errors in quantitative estimates of perfusion-related variables. Using a look-up table approach, the image intensities can be corrected easily and rapidly before calculating the serial concentrations of Gd contrast agents in the AIF and myocardial wall; these corrected concentrations should provide more accurate inputs for tracer kinetics modeling for perfusion calculations.

## Funding

National Institutes of Health grant R01-HL083309.
